# P-2179. Successful Early Detection of the Enterovirus-D68 Outbreak among Children by Wastewater Surveillance in Niigata City, Japan, 2024

**DOI:** 10.1093/ofid/ofaf695.2342

**Published:** 2026-01-11

**Authors:** Jun Tachikawa, Yuta Aizawa, R I e Habuka, Kotaro Tsushima, Nur Irma Safitri, Tatsuki Ikuse, Masaaki Kitajima, Akihiko Saitoh

**Affiliations:** Department of Pediatrics , Niigata University Graduate School of Medical and Dental Sciences, Niigata, Japan, Niigata, Niigata, Japan; Niigata University Graduate School of Medical and Dental Sciences, Niigata, Niigata, Japan; Niigata Univeisty, Niigata, Niigata, Japan; Niigata Univeisty, Niigata, Niigata, Japan; Niigata Univeisty, Niigata, Niigata, Japan; Niigata University, Niigata, Niigata, Japan; The University of Tokyo, Tokyo, Tokyo, Japan; Niigata University, Niigata, Niigata, Japan

## Abstract

**Background:**

Enterovirus-D68(EV-D68) causes acute asthma-like respiratory illness in children. This re-emerging virus is classified into four clades (A–D) based on the sequence of the viral protein 1 (VP1) region. Clade B strains are predominantly isolated from children, and clade D strains are more frequently detected from adults. Prediction of the next EV-D68 outbreak has become more challenging following the pandemic of the coronavirus disease 2019 (COVID-19). Recently, wastewater surveillance has provided community-level monitoring and has shown potential for predicting outbreaks of viral diseases, including the COVID-19.

Mean concentration of enterovirus-D68 (EV-D68) RNA in wastewater from two wastewater treatment plants and weekly number of admitted pediatric patients with wheezing episodes in Niigata City, Japan, 2024

**Figure:**
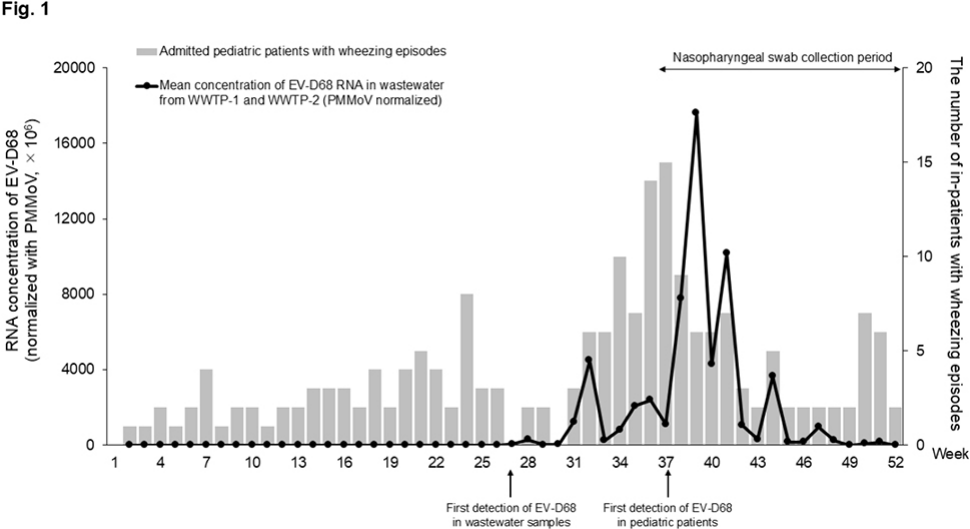
Black line indicates the mean concentration of enterovirus-D68 RNA in wastewater from two wastewater treatment plants (normalized with pepper mild mottle virus). Grey bars indicate the weekly number of children with wheezing episodes who were admitted to 6 hospitals in Niigata City, Japan, in 2024. Nasopharyngeal swab samples were collected from the admitted patients during week 37–52 of 2024. The first detection of EV-D68 in wastewater samples and pediatric patients was in week 27 and week 37, respectively.

**Methods:**

Influent wastewater was collected once a week from two wastewater treatment plants (WWTP) in Niigata, Japan during January–December 2024. EV-D68 RNA was detected by using the Efficient and Practical virus Identification System with Enhanced Sensitivity for Membrane (EPISENS-M) method. Concentrations of EV-D68 RNA were normalized to those of pepper mild mottle virus (PMMoV). Following the detection of EV-D68 in wastewater samples, and an increase in pediatric hospitalizations for asthma-like respiratory illness was recognized by pediatricians in Niigata City, nasopharyngeal (NP) swab samples were collected from children admitted with wheezing episodes. VP1 Sanger sequencing and phylogenetic analysis were performed on EV-D68 positive samples. The number of admitted pediatric patients with wheezing episodes was also collected from 6 hospitals in Niigata City.

**Results:**

EV-D68 RNA was detected in wastewater from week 27 (July), peaking in week 39 (September). Pediatric wheezing admissions peaked in week 37 (September), 2024. Wastewater EV-D68 RNA concentrations correlated with pediatric admissions (r = 0.48, P < 0.001). (Figure 1) Of 31 NP swab samples collected from weeks 37-52 (September-December), 16 (51.6%) were EV-D68 positive. All EV-D68 strains detected from NP swabs were in clade B3, whereas strains detected from the wastewater included clades B3 and D1.

**Conclusion:**

Wastewater surveillance enabled the early detection of an EV-D68 outbreak among children. Additionally, it was also useful to identify the dominant clades of EV-D68 in the community. The information obtained from the study could be used for predicting future outbreaks of EV-D68.

**Disclosures:**

Masaaki Kitajima, Dr.Eng., AdvanSentinel Inc.: Grant/Research Support|Shimadzu Corporation: Grant/Research Support|Shionogi & Co., Ltd.: Grant/Research Support|Shionogi & Co., Ltd.: Receive patent royalties .

